# Enhancement of Optomechanical Squeezing of Light Using the Optical Coherent Feedback

**DOI:** 10.3390/e24121741

**Published:** 2022-11-29

**Authors:** Zhenhua Wu, Zhen Yi, Wenju Gu, Lihui Sun, Zbigniew Ficek

**Affiliations:** 1 School of Physics and Optoelectronic Engineering, Yangtze University, Jingzhou 434023, China; 2 Quantum Optics and Engineering Division, Institute of Physics, University of Zielona Góra, Szafrana 4a, 65-516 Zielona Góra, Poland

**Keywords:** coherent feedback, optomechanical squeezing of light, optical and mechanical losses

## Abstract

A coherent feedback scheme is used to enhance the degree of squeezing of the output field in a cavity optomechanical system. In the feedback loop, a beam splitter (BS) plays the roles of both a feedback controller and an input–output port. To realize effective enhancement, the output quadrature should take the same form as the input quadrature, and the system should operate at the deamplification situation in the meantime. This can be realized by choosing an appropriate frequency-dependent phase angle for the generalized quadrature. Additionally, both the transmissivity of the BS and the phase factor induced by time delays in the loop affect optical squeezing. For the fixed frequency, the optimal values of transmissivity and phase factor can be used to achieve the enhanced optical squeezing. The effect of optical losses on squeezing is also discussed. Optical squeezing is degraded by the introduced vacuum noise owing to the inefficient transmission in the loop. We show that the enhancement of squeezing is achievable with the parameters of the current experiments.

## 1. Introduction

Squeezed states were first suggested to increase the displacement sensitivity of large-scale gravitational-wave detections [1,2,3]. The squeezed states of light have reduced quantum noise in one of their quadrature phase components compared with coherent states. This property led the squeezed states of light being found useful in optical communication to improve the channel capacity [4,5], fault-tolerant measurement-based quantum computation [6], metrology in more applied settings [7,8], etc. After the first generation of squeezed states in the nondegenerate four-wave mixing [9], the states were soon realized in the other platforms, such as optical fibers [10] and nonlinear crystals [11]. With the technical advancements, including the successful development of low-noise electronics, low-loss optical components, and high-efficiency photon diodes, substantial improvement in the squeezing level has been achieved in modern experiments. Since the benchmark 10 dB squeezing limit was exceeded in 2008 [12], further increases in squeezed levels have been achieved in subsequent experiments [13,14,15]. In addition to these nonlinear systems, cavity optomechanical systems, in which a cavity couples to a mechanical oscillator by the radiation pressure force, have been developed to manipulate the quantum fluctuations of light [16]. When the radiation pressure is sufficiently strong, quantum fluctuations become the dominant driving force on the oscillator. The optomechanical interaction can generate correlations between the oscillator and the cavity field. The correlations could be employed to squeeze the output field from the interferometer, which can reach below the shot-noise level. However, fluctuations in the conjugate quadrature are increased [17,18]. In comparison to the commonly used nonlinear optical media, optomechanical squeezing has a number of advantages: independent of the optical wavelength in the range from microwaves to optical fields [19,20], an adjustable frequency dependence of the squeezing quadrature using optical spring effects [21,22], and great capacity to be miniaturized in the long term [23].

A possibility to achieve light with significantly squeezed fluctuations stimulates further applications. For instance, the strong and broadband squeezed light source is required in the audio-frequency band of GW detection [22]. Thus, numerous methods have been exploited to increase the squeezing level in the nonlinear optical systems [24,25,26]. Apart from nonlinear optical systems, a large body of research papers have focused on achieving significant squeezing of fluctuations in optomechanical systems [27,28,29,30,31]. However, how to enhance the optomechanical squeezing of light is still difficult. For state-of-the-art optomechanical settings, such as an ensemble of ultracold atoms in an optical cavity [32], a micromechanical resonator coupled to a nanophotonic cavity [33], a high vibrating membrane in the middle of an optical cavity [34], and a single-crystal micro-cantilever as an output mirror [22], the maximum squeezing level only reaches 32% (1.7dB), below the shot-noise limit. Therefore, several key challenges for optomechanically squeezed states remain in practice. The methods used in nonlinear optical systems can be adopted to improve the squeezing level and bandwidth in cavity optomechanics, such as cascading systems and utilizing feedback loops [35,36]. Here we study the optimal condition to achieve the enhancement of optomechanical squeezing of light with the use of optical coherent feedback. Distinct from the measurement-based feedback control [37], the controller in the coherent feedback loop is a quantum system. It can modulate and feed back the output to control the system in a coherent manner [38,39,40,41,42,43]. In the loop, no measurement is performed, and thus no excess measurement backaction noise is introduced into the system. Owing to this feature, coherent feedback control is useful to deal with noise reduction problems, which is the key issue in control theory. For example, backaction noise cancellation based on direction interaction, proposed by Tsang and Caves [44], can be equivalently realized by the coherent feedback control [45]. A comprehensive review on the measurement-based feedback and the coherent feedback is presented in reference [45].

In this paper, we propose a method to increase the degree of squeezing and bandwidth of optical states in a cavity optomechanical system via utilizing the coherent feedback scheme. We are particularly focused on the role of a passive feedback loop which corresponds to interferometric processes involving a BS and losses [46]. The BS plays the role of both a feedback controller and an input–output port. It is kind of analogous to the signal-recycled Michelson interferometer, where a signal-recycling mirror with very high reflectivity is utilized [47,48]. However, there exist some differences. Coherent feedback is a branch of quantum feedback control theory and particularly focuses on the optimal control to achieve the control goal. We show that the output field quadrature from the cavity optomechanical system generally contains both the squeezed and its conjugate anti-squeezed quadratures. We found that the presence of the anti-squeezed quadrature has a destructive effect on squeezing in the feedback loop because of its enhanced noise. In order to avoid the effect of that enhanced noise, we propose to consider the generalized output quadrature which is deamplified and takes the same form as the input quadrature. This form of the output quadrature can be achieved by varying the frequency-dependent phase angle of the generalized quadrature. Additionally, both the transmissivity of BS and phase factor induced by time delays in the loop contribute to optical squeezing. For the fixed frequency, the optimal values of transmissivity and phase factor can be utilized to achieve the maximum enhancement of the optical squeezing. We also discuss the influence of losses on squeezing, including the cavity and the oscillator. We found that cavity losses can degrade the squeezing because of the introduction of noises, but the mechanical losses have a negligible effect on the optical squeezing due to the ultra-high Q factor in the current experiments.

This paper is structured as follows. In Section 2, the cavity optomechanical system assisted by the coherent feedback loop is introduced. In Section 3, the squeezing spectrum of the generalized output quadrature is derived, and the enhancement of squeezing with the optimal condition of coherent feedback is investigated. In Section 4, the influences of optical and mechanical losses are discussed. A brief summery of our results and conclusions is given in Section 5.

## 2. Cavity Optomechanical System Assisted by the Coherent Feedback

**Figure 1:**
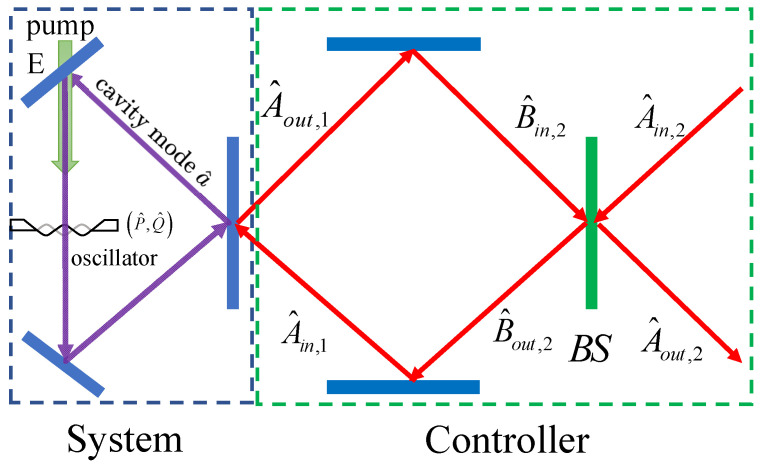
Illustration of a cavity optomechanical system with the use of a coherent feedback system. A beam splitter (BS) plays the role of both a feedback controller and an input–output port.

We consider a scheme to improve the optomechanical squeezing of light with the use of the coherent feedback control, as shown in Figure 1. The optomechanical system consists of an optical cavity and a mechanical oscillator. The coherent feedback loop is constituted by a BS and two reflecting mirrors. The BS also serves as the input–output port. The optomechanical system is described by the Hamiltonian

(1) $$\begin{array}{cc} \hat{H} & =\hbar \omega_{c}{\hat{a}}^{†}\hat{a}+\frac{1}{2}\hbar \omega_{m}\left({\hat{Q}}^{2}+{\hat{P}}^{2}\right)+\hbar g_{0}{\hat{a}}^{†}\hat{a}\hat{Q}, \end{array}$$

where $\omega_{c}$ and $\omega_{m}$ are the optical and mechanical resonance frequencies, respectively. Here, $\hat{a}$ is the intracavity annihilation operator, $\hat{Q}$ and $\hat{P}$ are the mechanical position and momentum operators and $g_{0}$ is the single-photon optomechanical coupling strength. In order to achieve the strong effective optomechanical coupling, the cavity field is pumped by a strong laser field. The pumped Hamiltonian is described by

(2) $$\begin{array}{c} {\hat{H}}_{p}=i\hbar \left(E{\hat{a}}^{†}e^{i\omega_{l}t}-E^{*}\hat{a}e^{-i\omega_{l}t}\right), \end{array}$$

in which *E* is the driven strength and $\omega_{l}$ is the laser frequency. In the common linearization treatment of the nonlinear optomechanical interaction [16], the optical field operator is written as the sum of its expectation value and a fluctuation operator, $\hat{a}=\left(\bar{a}+\hat{c}\right)e^{i\omega_{l}t}$, where $\bar{a}=\langle \hat{a}\rangle$ denotes the amplitude of the intracavity field and $\hat{c}$ denotes the fluctuation operator. The amplitude $\bar{a}$ can be supposed to be real by adjusting the phase of the pump field. After the linearization treatment and moving to the rotating frame of the laser’s frequency, the Hamiltonian becomes

(3) $$\begin{array}{cc} \hat{H} & =-\hbar \Delta {\hat{c}}^{†}\hat{c}+\frac{1}{2}\hbar \omega_{m}\left({\hat{Q}}^{2}+{\hat{P}}^{2}\right)+\hbar g\left({\hat{c}}^{†}+\hat{c}\right)\hat{Q} \end{array}$$

where $\Delta =\omega_{l}-\omega_{c}$ is the detuning between the laser and the cavity frequencies, and $g=g_{0}\bar{c}$ is the effectively enhanced optomechanical coupling strength.

Taking into the consideration damping rates of the cavity field and the oscillator and their noise, the equations of motion for the operators are of the form

(4) $$\begin{array}{cc} \; & \frac{d}{dt}\hat{Q}=\omega_{m}\hat{P},\\ \; & \frac{d}{dt}\hat{P}=-\omega_{m}\hat{Q}-g\left({\hat{c}}^{†}+\hat{c}\right)-\Gamma_{0}\hat{P}+\sqrt{\Gamma_{0}}\hat{\xi },\\ \; & \frac{d}{dt}\hat{c}=\left(i\Delta -\frac{\kappa }{2}\right)\hat{c}-ig\hat{Q}+\sqrt{\kappa }{\hat{A}}_{\mathrm{in},1},\\ \; & \frac{d}{dt}{\hat{c}}^{†}=-\left(i\Delta +\frac{\kappa }{2}\right){\hat{c}}^{†}+ig\hat{Q}+\sqrt{\kappa }{\hat{A}}_{\mathrm{in},1}^{†}. \end{array}$$

where κ and $\Gamma_{0}$ are damping rates of the optical and mechanical modes, respectively; and $\hat{\xi }$ is the mechanical thermal noise which obeys the correlations $\langle \hat{\xi }\left(\tau \right)\hat{\xi }\left(\tau^{\prime }\right)\rangle =\left(n_{\mathrm{th}}+1/2\right)\delta \left(\tau -\tau^{\prime }\right)$, where $n_{\mathrm{th}}=1/\left[e^{\hbar \omega_{m}/k_{B}T_{m}}-1\right]$ is the number of thermal photons, $k_{B}$ is the Boltzmann constant and $T_{m}$ is the temperature. The operator ${\hat{A}}_{\mathrm{in},1}$ stands for the optical noise in the cavity mode.

For the coherent feedback system, proper choice of the BS is essential to improve the optomechanical squeezing of light. The input field ${\hat{A}}_{\mathrm{in},2}\left(t\right)$ is sent to one port of the BS, and then one of its outputs ${\hat{B}}_{\mathrm{out},2}\left(t\right)$ is sent to the optomechanical cavity. The output field of optomechanical cavity ${\hat{A}}_{\mathrm{out},1}\left(t\right)$ is sent back to the optomechanical cavity to form a closed loop. Finally, the target output field $A_{\mathrm{out},2}\left(t\right)$ will be achieved at the other output of the BS. The input–output relations at the BS are given by

(5) $$\begin{array}{cc} \; & {\hat{A}}_{\mathrm{out},2}\left(t\right)=\sqrt{1-T_{2}}{\hat{A}}_{\mathrm{in},2}\left(t\right)+\sqrt{T_{2}}{\hat{B}}_{\mathrm{in},2}\left(t\right),\\ \; & {\hat{B}}_{\mathrm{out},2}\left(t\right)=-\sqrt{1-T_{2}}{\hat{B}}_{\mathrm{in},2}\left(t\right)+\sqrt{T_{2}}{\hat{A}}_{\mathrm{in},2}\left(t\right), \end{array}$$

where $T_{2}$ is the transmissivity of the BS. Further, time delays in the feedback loop should be taken into account. Therefore, we introduce time delays $\tau_{a}=l_{a}/c$ and $\tau_{b}=l_{b}/c$ generated by the optical path lengths $l_{a}$ and $l_{b}$, where $l_{a}$ is the path length from the BS to the optomechanical cavity and $l_{b}$ is the path length from the optomechanical cavity to the BS. Hence, we have

(6) $$\begin{array}{cc} \; & {\hat{A}}_{\mathrm{in},1}\left(t\right)={\hat{B}}_{\mathrm{out},2}\left(t-\tau_{a}\right),\\ \; & {\hat{B}}_{\mathrm{in},2}\left(t\right)={\hat{A}}_{\mathrm{out},1}\left(t-\tau_{b}\right). \end{array}$$

In addition, at the output mirror of the optomechanical cavity, the output field ${\hat{A}}_{\mathrm{out},1}\left(t\right)$ fulfills the input–output relation

(7) $$\begin{array}{c} {\hat{A}}_{\mathrm{out},1}\left(t\right)=-{\hat{A}}_{\mathrm{in},1}\left(t\right)+\sqrt{\kappa }\hat{c}\left(t\right). \end{array}$$

In order to obtain the spectrum of the output noise, the Langevin Equation (4) can be solved in the frequency domain using Fourier transform $\hat{F}\left[\omega \right]=\frac{1}{\sqrt{2\pi }}\int_{-\infty }^{\infty }\hat{F}\left(t\right)e^{i\omega t}dt$ and ${\hat{F}}^{†}\left[\omega \right]=\frac{1}{\sqrt{2\pi }}\int_{-\infty }^{\infty }{\hat{F}}^{†}\left(t\right)e^{i\omega t}dt$. The frequency-dependent operators of the optical and mechanical modes are

(8) $$\begin{array}{cc} \; & \hat{Q}\left[\omega \right]=\left[-g\left({\hat{c}}^{†}\left[\omega \right]+\hat{c}\left[\omega \right]\right)+\sqrt{\Gamma_{0}}\hat{\xi }\left[\omega \right]\right]\chi_{m}\left[\omega \right],\\ \; & \hat{c}\left[\omega \right]=\left(-ig\hat{Q}\left[\omega \right]+\sqrt{\kappa }{\hat{A}}_{\mathrm{in},1}\left[\omega \right]\right)\chi_{c}\left[\omega \right],\\ \; & {\hat{c}}^{†}\left[\omega \right]=\left(ig\hat{Q}\left[\omega \right]+\sqrt{\kappa }{\hat{A}}_{\mathrm{in},1}^{†}\left[\omega \right]\right)\chi_{c}^{*}\left[-\omega \right], \end{array}$$

where $\chi_{m}\left[\omega \right]=\omega_{m}{\left(\omega_{m}^{2}-\omega^{2}-i\Gamma_{0}\omega \right)}^{-1}$ and $\chi_{c}\left[\omega \right]=1/\left[-i\left(\Delta +\omega \right)+\frac{\kappa }{2}\right]$ are the mechanical and cavity susceptibilities, respectively.

From the input–output relation (7) and Equation (8), we can see that the output field amplitude ${\hat{A}}_{\mathrm{out},1}\left[\omega \right]$ is related to the input field amplitude and the oscillator’s position by

(9) $$\begin{array}{c} {\hat{A}}_{\mathrm{out},1}\left[\omega \right]=-ig\sqrt{\kappa }\chi_{c}\left[\omega \right]\hat{Q}\left[\omega \right]+\left(\kappa \chi_{c}\left[\omega \right]-1\right){\hat{A}}_{\mathrm{in},1}\left[\omega \right]. \end{array}$$

Thus, the output field contains the information about the oscillator’s motion. The oscillator’s position operator $\hat{Q}\left[\omega \right]$ is of the form

(10) $$\begin{array}{c} \hat{Q}\left[\omega \right]=\chi_{m}^{\mathrm{eff}}\left[\omega \right]\left({\hat{F}}_{\mathrm{QBA}}\left[\omega \right]+\sqrt{\Gamma_{0}}\hat{\xi }\left[\omega \right]\right), \end{array}$$

where $\chi_{m}^{\mathrm{eff}}\left[\omega \right]={\left[\chi_{c}{\left[\omega \right]}^{-1}-ig^{2}\left(\chi_{c}\left[\omega \right]-\chi_{c}^{*}\left[-\omega \right]\right)\right]}^{-1}$ denotes the effective susceptibility of the oscillator, which has been altered by the optomechanical interaction. The quantum backaction force ${\hat{F}}_{\mathrm{QBA}}\left[\omega \right]$ appearing in Equation (10) is caused by the radiation pressure shot noise and is of the form

(11) $$\begin{array}{c} {\hat{F}}_{\mathrm{QBA}}\left[\omega \right]=-g\sqrt{\kappa }\left(\chi_{c}^{*}\left[-\omega \right]{\hat{A}}_{\mathrm{in},1}^{†}\left[\omega \right]+\chi_{c}\left[\omega \right]{\hat{A}}_{\mathrm{in},1}\left[\omega \right]\right) \end{array}$$

The destructive interference of the quantum backaction force and mechanical position fluctuations will give rise to the squeezing of the output cavity field [33,49].

## 3. Derivation of Squeezing Spectrum

In order to derive the expression for the squeezing spectrum of the output field, we introduce generalized quadratures

(12) $$\begin{array}{cc} \; & {\hat{X}}_{\mathrm{out},m}^{\theta }\left[\omega \right]=\left({\hat{A}}_{\mathrm{out},m}^{†}\left[\omega \right]e^{i\theta }+{\hat{A}}_{\mathrm{out},m}\left[\omega \right]e^{-i\theta }\right)/2,\\ \; & {\hat{Y}}_{\mathrm{out},m}^{\theta }\left[\omega \right]=i\left({\hat{A}}_{\mathrm{out},m}^{†}\left[\omega \right]e^{i\theta }-{\hat{A}}_{\mathrm{out},m}\left[\omega \right]e^{-i\theta }\right)/2, \end{array}$$

with the quadrature angle θ and m=1,2. Using Equations (9) and (10), we found that the quadrature component ${\hat{X}}_{\mathrm{out},1}^{\theta }\left[\omega \right]$ of the output optomechanical cavity field is related to the input quadratures ${\hat{X}}_{\mathrm{in},1}^{\theta }\left[\omega \right]$ and ${\hat{Y}}_{\mathrm{in},1}^{\theta }\left[\omega \right]$ by

(13) $$\begin{array}{c} {\hat{X}}_{\mathrm{out},1}^{\theta }\left[\omega \right]=C_{\mathrm{XX}}^{\theta }\left[\omega \right]{\hat{X}}_{\mathrm{in},1}^{\theta }\left[\omega \right]+C_{\mathrm{XY}}^{\theta }\left[\omega \right]{\hat{Y}}_{\mathrm{in},1}^{\theta }\left[\omega \right]+C_{X\xi }^{\theta }\left[\omega \right]\hat{\xi }\left[\omega \right], \end{array}$$

where the coefficients are

(14) $$\begin{array}{cc} C_{\mathrm{XX}}^{\theta }\left[\omega \right]= & \frac{1}{2}\kappa [\left(\chi_{c}^{*}\left[-\omega \right]+\chi_{c}\left[\omega \right]\right)-ig^{2}\left(\chi_{c}^{*2}\left[-\omega \right]-\chi_{c}^{2}\left[\omega \right]\right)\chi_{m}^{\mathrm{eff}}\left[\omega \right]\\ \; & +2\sin2\theta g^{2}\chi_{c}\left[\omega \right]\chi_{c}^{*}\left[-\omega \right]\chi_{m}^{\mathrm{eff}}\left[\omega \right]]-1,\\ C_{\mathrm{XY}}^{\theta }\left[\omega \right]= & -\frac{1}{2}i\kappa [\left(\chi_{c}^{*}\left[-\omega \right]-\chi_{c}\left[\omega \right]\right)-ig^{2}\left(\chi_{c}^{2}\left[\omega \right]+\chi_{c}^{*2}\left[-\omega \right]\right)\chi_{m}^{\mathrm{eff}}\left[\omega \right]\\ \; & +2\cos2\theta ig^{2}\chi_{c}\left[\omega \right]\chi_{c}^{*}\left[-\omega \right]\chi_{m}^{\mathrm{eff}}\left[\omega \right]],\\ C_{X\xi }^{\theta }\left[\omega \right]= & -\frac{1}{2}ig\sqrt{\kappa \Gamma_{0}}\chi_{m}^{\mathrm{eff}}\left[\omega \right]\left(\chi_{c}\left[\omega \right]e^{-i\theta }-\chi_{c}^{*}\left[-\omega \right]e^{i\theta }\right). \end{array}$$

We emphasize that an enhancement of squeezing via the coherent feedback loop can be achieved only when the quadrature of output field ${\hat{X}}_{\mathrm{out},1}^{\theta }\left[\omega \right]$ relates to the same quadrature of input field, i.e., ${\hat{X}}_{\mathrm{in},1}^{\theta }\left[\omega \right]$, without the contribution of the conjugate quadrature ${\hat{Y}}_{\mathrm{in},1}^{\theta }\left[\omega \right]$. Otherwise, the presence of the conjugate quadrature would be fed back to degrade the further squeezing in the loop. In the nonlinear crystal system, the output quadrature is just the input quadrature. The coherent feedback control works well to enhance optical field squeezing. Thus, in the optomechanical system, if we aim to achieve the enhanced squeezing in the quadrature ${\hat{X}}_{\mathrm{out},1}^{\theta }\left[\omega \right]$, the coefficient $C_{\mathrm{XY}}^{\theta }\left[\omega \right]$ in Equation (14) has to be zero. In addition, the coefficient $|C_{\mathrm{XX}}^{\theta }\left[\omega \right]|$ has to be less than one. To ensure that $C_{\mathrm{XY}}^{\theta }\left[\omega \right]=0$, the following condition has to be satisfied:

(15) $$\begin{array}{c} \chi_{m}^{-1}\left[\omega \right]\Delta +2g^{2}\sin^{2}\theta =0. \end{array}$$

The above condition is difficult to achieve because it is a complex equation. However, if $\Gamma_{0}$ can be neglected for now, the equation can be fulfilled with the phase angle θ given by

(16) $$\begin{array}{c} \sin\theta =\pm \sqrt{-\frac{\Delta (\omega_{m}^{2}-\omega^{2})}{2g^{2}\omega_{m}}}, \end{array}$$

Note that the phase angle depends on frequency. Homodyne detection of the output field with the frequency-dependent homodyne phase is realizable, e.g., through a variational-output interferometer [3]. In practice, the mechanical factor reaches values up to $Q=\omega_{m}/\Gamma_{0}=10^{9}$ in the platform using the soft clamping technique [49,50,51]. In this case, the condition for squeezing $|C_{\mathrm{XX}}^{\theta }\left[\omega \right]|<1$ should be fulfilled. This is realizable by choosing a positive or negative value of sinθ.

Subject to the conditions (15) and (16), and introducing ${\tilde{C}}_{\mathrm{XX}}^{\theta }\left[\omega \right]=C_{\mathrm{XX}}^{\theta }\left[\omega \right]e^{i\psi }$ with the phase factor $\psi =\omega (\tau_{a}+\tau_{b})$ caused by time delays in the loop, we can obtain the final output quadrature ${\hat{X}}_{\mathrm{out},2}^{\theta }\left[\omega \right]$ as

(17) $$\begin{array}{c} {\hat{X}}_{\mathrm{out},2}^{\theta }\left[\omega \right]=\left(\frac{\sqrt{1-T_{2}}+{\tilde{C}}_{\mathrm{XX}}^{\theta }\left[\omega \right]}{1+\sqrt{1-T_{2}}{\tilde{C}}_{\mathrm{XX}}^{\theta }\left[\omega \right]}\right){\hat{X}}_{\mathrm{in},2}^{\theta }\left[\omega \right]+\frac{\sqrt{T_{2}}C_{X\xi }^{\theta }\left[\omega \right]e^{i\omega \tau_{b}}}{1+\sqrt{1-T_{2}}{\tilde{C}}_{\mathrm{XX}}^{\theta }\left[\omega \right]}\hat{\xi }\left[\omega \right]. \end{array}$$

The first term indicates the squeezing of the input field quadrature, and the second terms indicates the influence of mechanical thermal noise.

Having an available quadrature component of the the output field, we can evaluate the squeezing spectrum defined by $S_{{\hat{X}}_{\mathrm{out},2}^{\theta }}\left[\omega \right]\delta \left(\omega +\omega^{\prime }\right)=\langle {\hat{X}}_{\mathrm{out},2}^{\theta }\left[\omega \right]{\hat{X}}_{\mathrm{out},2}^{\theta }\left[\omega^{\prime }\right]\rangle$. The condition for squeezing in the quadrature component is $S_{{\hat{X}}_{\mathrm{out},2}^{\theta }}\left[\omega \right]<\frac{1}{4}$. We found

(18) $$\begin{array}{c} S_{{\hat{X}}_{\mathrm{out},2}^{\theta }}\left[\omega \right]=\frac{1}{4}{\left|\frac{\sqrt{1-T_{2}}+{\tilde{C}}_{\mathrm{XX}}^{\theta }\left[\omega \right]}{1+\sqrt{1-T_{2}}{\tilde{C}}_{\mathrm{XX}}^{\theta }\left[\omega \right]}\right|}^{2}+{\left|\frac{\sqrt{T_{2}}C_{X\xi }^{\theta }\left[\omega \right]}{1+\sqrt{1-T_{2}}{\tilde{C}}_{\mathrm{XX}}^{\theta }\left[\omega \right]}\right|}^{2}\left(n_{\mathrm{th}}+\frac{1}{2}\right). \end{array}$$

Here both the transmissivity $T_{2}$ and the phase factor induced by time delays appear in ${\tilde{C}}_{\mathrm{XX}}^{\theta }\left[\omega \right]$, and they are tunable in the coherent feedback loop. In what follows, we demonstrate how to achieve the enhancement of squeezing through the optimal choice of these parameters.

### Enhancement of Squeezing with the Optimal Coherent Feedback

**Figure 2:**
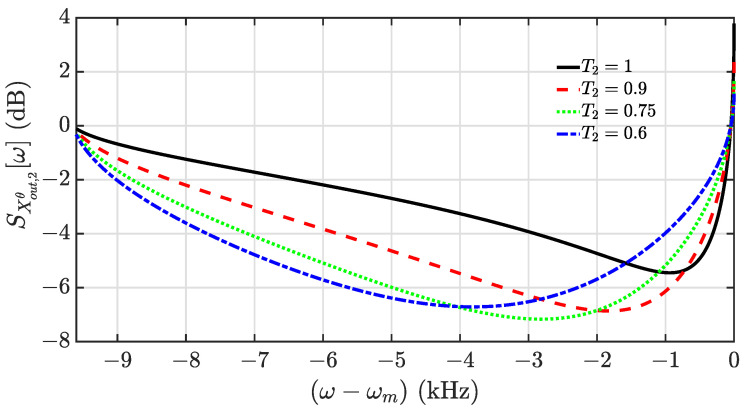
Squeezing spectra of the output quadrature ${\hat{X}}_{\mathrm{out},2}^{\theta }\left[\omega \right]$ plotted as a function of the frequency for different values of the transmissivity $T_{2}$: $T_{2}=1$ (black solid), $T_{2}=0.9$ (red dashed), $T_{2}=0.75$ (green dotted), and $T_{2}=0.6$ (blue dash-dotted). The detuning is Δ=−0.1κ. The other parameters are κ=2π×16.2MHz, $\omega_{m}=2\pi \times 1.135\;\mathrm{MHz}$, g=2π×50kHz, $Q=1.03\times 10^{9}$, $T_{m}=10\;K$, and ψ=π.

To clearly present the improvement of the squeezing level and the bandwidth with the use of the coherent feedback loop, we numerically analyze the spectrum. For the choices of values of the optomechanical parameters, we follow the experimental setup in reference [49], in which κ=2π×16.2MHz, g=2π×50kHz, $\omega_{m}=2\pi \times 1.135\;\mathrm{MHz}$, $Q=1.03\times 10^{9}$, and $T_{m}=10\;K$. Additionally, under the constraint of the phase angle θ in Equation (16), the frequency range of squeezing is related to the detuning Δ. For example, we could take Δ=−0.1κ to achieve the squeezing below the frequency of the oscillator, i.e., $\omega <\omega_{m}$. The positive detuning should be taken into account, such as Δ=0.1κ, for the squeezing above the frequency of the oscillator, i.e., $\omega >\omega_{m}$. In Figure 2, we numerically plot the squeezing spectra $S_{{\hat{X}}_{\mathrm{out},2}^{\theta }}\left[\omega \right]$ as a function of frequency for several different values of transmissivity $T_{2}$, under the assumption of ψ=π. When $T_{2}=1$, it is the case of squeezing without the coherent feedback. It is seen that the coherent feedback begins to work with $T_{2}<1$. This clearly shows that the coherent feedback can improve the squeezing performance of the output field compared to that of the uncontrolled case, including the squeezing level and bandwidth.

**Figure 3:**
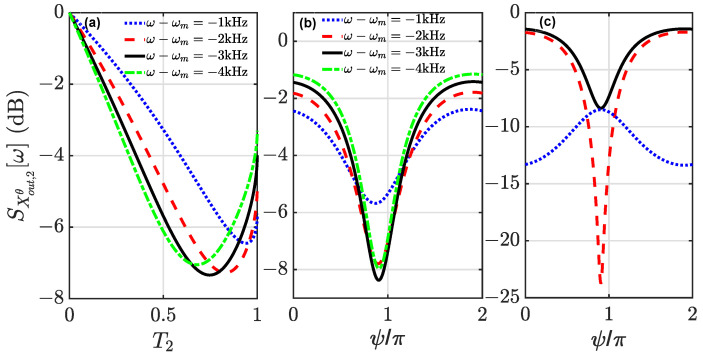
Squeezing spectra of the output field ${\hat{X}}_{\mathrm{out},2}^{\theta }\left[\omega \right]$ plotted in (**a**) as a function of the transmissivity $T_{2}$ for ψ=π and in (**b**) as a function of the phase factor ψ for $T_{2}=0.75$. In both plots, $\omega -\omega_{m}=\;-1$ kHz (blue dotted), $\omega -\omega_{m}=\;-2$ kHz (red dashed), $\omega -\omega_{m}=\;-3$ kHz (black solid), $\omega -\omega_{m}=\;-4$ kHz (green dash-dotted). In (**c**), contributions from the input light quadrature (red-dashed) and the mechanical thermal noise (blue dotted) on the optical squeezing (black solid) are presented. The other parameters are the same as in Figure 2.

We further explore the optimal conditions on the optical squeezing, including the transmissivity $T_{2}$ and the phase factor induced by times delays in the feedback loop. For the contribution from the input light quadrature in Equation (18), the optimal squeezing occurs under the condition $\sqrt{1-T_{2}}+{\tilde{C}}_{\mathrm{XX}}^{\theta }\left[\omega \right]=0$. Then, the squeezing is limited by the mechanical thermal noise. For the case of phase shift ψ=π, it means destructive interference. The squeezing as a function of $T_{2}$ at the fixed frequencies is plotted in Figure 3a. Obviously, there exists an optimal value of $T_{2}$ to reach a better squeezing level in comparison to that of the case without coherent feedback ($T_{2}=1$). The optimal value equals T2=1−Re[CXXθ[ω]]2 because $C_{XX}^{\theta }\left[\omega \right]$ is a complex number. This can be numerically validated with the parameters in Figure 3a. More accurately, the phase shift at the output mirror of the system due to the optomechanical interaction should be also taken into account. The phase angle of $C_{XX}^{\theta }\left[\omega \right]$ is about 0.1π for the given parameters. Then, the optimal phase factor should be ψ=0.9π to fulfill the destructive interference. In Figure 3b, we numerically present how the squeezing level depends on the phase factor at the fixed frequencies with $T_{2}=0.75$. The better squeezing levels can be observed at ψ=0.9π for all of the fixed frequencies. Additionally, the maximun squeezing level is 8dB here. In Figure 3c, the contributions from the input light quadrature and the mechanical thermal noise are presented, respectively. It clearly shows that the squeezing is limited by the mechanical thermal noise under the optimal conditions ($T_{2}=0.75$ and ψ=0.9π). Thus, the smaller $T_{m}/Q$ is, the better the squeezing becomes.

## 4. Effects of Optical and Mechanical Losses on Optical Squeezing

Optical loss is inevitable in the coherent feedback loop in practice, and it is generally modelled as a beam-splitter mixing:

(19) $$\begin{array}{cc} \; & {\hat{A}}_{\mathrm{in},1}\left[\omega \right]=\sqrt{\eta_{1}}{\hat{B}}_{\mathrm{out},2}\left[\omega \right]e^{i\omega \tau_{a}}+\sqrt{1-\eta_{1}}{\hat{a}}_{v_{1}}\left[\omega \right],\\ \; & {\hat{B}}_{\mathrm{in},2}\left[\omega \right]=\sqrt{\eta_{2}}{\hat{A}}_{\mathrm{out},1}\left[\omega \right]e^{i\omega \tau_{b}}+\sqrt{1-\eta_{2}}{\hat{a}}_{v_{2}}\left[\omega \right], \end{array}$$

where $\eta_{1}$ and $\eta_{2}$ are the transmission efficiencies in two paths, and ${\hat{a}}_{v_{1}}\left[\omega \right]$ and ${\hat{a}}_{v_{2}}\left[\omega \right]$ are the uncorrelated vacuum noises in the paths. Then, in the case of ψ=π, the output quadrature ${\hat{X}}_{\mathrm{out},2}^{\theta }\left[\omega \right]$ takes the form

(20) $$\begin{array}{c} {\hat{X}}_{\mathrm{out},2}^{\theta }\left[\omega \right]=\Xi_{\mathrm{XX}}\left[\omega \right]{\hat{X}}_{\mathrm{in},2}^{\theta }\left[\omega \right]+\Xi_{{\mathrm{Xv}}_{1}}\left[\omega \right]{\hat{X}}_{v_{1}}^{\theta }\left[\omega \right]+\Xi_{{\mathrm{Xv}}_{2}}\left[\omega \right]{\hat{X}}_{v_{2}}^{\theta }\left[\omega \right]+\Xi_{X\xi }\left[\omega \right]\hat{\xi }\left[\omega \right], \end{array}$$

where the quadratures of the vacuum noises are ${\hat{X}}_{v_{1}}^{\theta }\left[\omega \right]=\left({\hat{a}}_{v_{1}}\left[\omega \right]e^{-i\theta }+{\hat{a}}_{v_{1}}^{†}\left[\omega \right]e^{i\theta }\right)/2$ and ${\hat{X}}_{v_{2}}^{\theta }\left[\omega \right]=\left({\hat{a}}_{v_{2}}\left[\omega \right]e^{-i\theta }+{\hat{a}}_{v_{2}}^{†}\left[\omega \right]e^{i\theta }\right)/2$, and the coefficients are

(21) $$\begin{array}{cc} \Xi_{\mathrm{XX}}\left[\omega \right]= & \sqrt{1-T_{2}}-\frac{\sqrt{\eta_{1}\eta_{2}}T_{2}C_{XX}^{\theta }\left[\omega \right]}{1-\sqrt{\eta_{1}\eta_{2}}\sqrt{1-T_{2}}C_{\mathrm{XX}}^{\theta }\left[\omega \right]},\\ \Xi_{{\mathrm{Xv}}_{1}}\left[\omega \right]= & \frac{\sqrt{1-\eta_{1}}\sqrt{\eta_{2}}\sqrt{T_{2}}C_{\mathrm{XX}}^{\theta }\left[\omega \right]e^{i\omega \tau_{b}}}{1-\sqrt{\eta_{1}\eta_{2}}\sqrt{1-T_{2}}C_{\mathrm{XX}}^{\theta }\left[\omega \right]},\\ \Xi_{{\mathrm{Xv}}_{2}}\left[\omega \right]= & \sqrt{1-\eta_{1}}\sqrt{T_{2}}+\frac{\sqrt{\eta_{1}\eta_{2}}\sqrt{1-\eta_{2}}\sqrt{T_{2}}\sqrt{1-T_{2}}C_{\mathrm{XX}}^{\theta }\left[\omega \right]}{1-\sqrt{\eta_{1}\eta_{2}}\sqrt{1-T_{2}}C_{\mathrm{XX}}^{\theta }\left[\omega \right]},\\ \Xi_{X\xi }\left[\omega \right]= & \frac{\sqrt{\eta_{2}}\sqrt{T_{2}}C_{X\xi }^{\theta }\left[\omega \right]e^{i\omega \tau_{b}}}{1-\sqrt{\eta_{1}\eta_{2}}\sqrt{1-T_{2}}C_{\mathrm{XX}}^{\theta }\left[\omega \right]}. \end{array}$$

In this case, the spectrum of the output quadrature is given by

(22) $$\begin{array}{cc} S_{{\hat{X}}_{\mathrm{out},2}^{\theta }}\left[\omega \right]= & \frac{1}{4}\left({\left|\Xi_{\mathrm{XX}}\left[\omega \right]\right|}^{2}+{\left|\Xi_{{\mathrm{Xv}}_{1}}\left[\omega \right]\right|}^{2}+{\left|\Xi_{{\mathrm{Xv}}_{2}}\left[\omega \right]\right|}^{2}\right)+{\left|\Xi_{X\xi }\left[\omega \right]\right|}^{2}\left(n_{\mathrm{th}}+\frac{1}{2}\right). \end{array}$$

**Figure 4:**
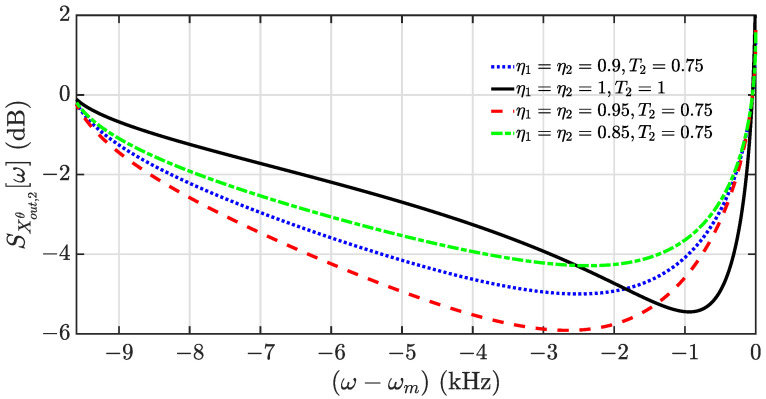
Squeezing spectra of the output field ${\hat{X}}_{\mathrm{out},2}^{\theta }\left[\omega \right]$ as a function of frequency for different values of transmissivity $T_{2}$ and transmission efficiencies. i.e., $\eta_{1}=\eta_{2}=0.9,T_{2}=0.75$ (blue dotted), $\eta_{1}=\eta_{2}=1,T_{2}=1$ (black solid), $\eta_{1}=\eta_{2}=0.95,T_{2}=0.75$ (red dashed), $\eta_{1}=\eta_{2}=0.85,T_{2}=0.75$ (green dash-dotted). The other parameters are the same as in Figure 2.

Since the optical loss in the feedback loop introduces the uncorrelated vacuum noise, the squeezing level is decreased as shown in Figure 4. In comparison to those of the case without the coherent feedback ($\eta_{1}=\eta_{2}=1$, $T_{2}=1$), a better squeezing level and boarder bandwidth can be still reached at $\eta_{1}=\eta_{2}=0.95$ and $T_{2}=0.75$. The higher propagation efficiency of the feedback loop above 0.95 has been demonstrated in the optical parametric oscillator platforms [52,53]. Moreover, even with $\eta_{1}=\eta_{2}=0.9$ and $T_{2}=0.75$, the squeezing range gets broader with the squeezing level that is close to the maximum squeezing level in the case without the coherent feedback. However, too much optical loss will limit the coherent feedback to achieve the enhancement of squeezing.

Recall that the condition of $C_{\mathrm{XY}}^{\theta }\left[\omega \right]=0$ is satisfied by ignoring the mechanical damping rate $\Gamma_{0}$ owing to the ultra-high mechanical Q factor. When $\Gamma_{0}$ is taken into account, the coefficient $C_{\mathrm{XY}}^{\theta }\left[\omega \right]$ becomes nonzero. However, for the given parameters [35], it is six orders of magnitude lower than $C_{XX}^{\theta }\left[\omega \right]$. When including the effect of the input of the anti-squeezed quadrature ${\hat{Y}}_{\mathrm{out},1}^{\theta }\left[\omega \right]$, the output field quadrature ${\hat{X}}_{\mathrm{out},2}^{\theta }\left[\omega \right]$ becomes

(23) $$\begin{array}{c} {\hat{X}}_{\mathrm{out},2}^{\theta }\left[\omega \right]=\Xi_{X}^{\prime }\left[\omega \right]{\hat{X}}_{\mathrm{in},2}\left[\omega \right]+\Xi_{Y}^{\prime }\left[\omega \right]{\hat{Y}}_{\mathrm{in},2}\left[\omega \right]+\Xi_{\xi }^{\prime }\left[\omega \right]\hat{\xi }\left[\omega \right], \end{array}$$

with the coefficients

(24) $$\begin{array}{cc} \Xi_{X}^{\prime }\left[\omega \right]= & \sqrt{1-T_{2}}-\frac{T_{2}C_{\mathrm{XX}}^{\theta }\left[\omega \right]+\frac{T_{2}\sqrt{1-T_{2}}C_{\mathrm{YX}}^{\theta }\left[\omega \right]C_{\mathrm{XY}}^{\theta }\left[\omega \right]}{1-\sqrt{1-T_{2}}C_{\mathrm{YY}}^{\theta }\left[\omega \right]}}{1-\sqrt{1-T_{2}}C_{\mathrm{XX}}^{\theta }\left[\omega \right]-\frac{\left(1-T_{2}\right)C_{\mathrm{YX}}^{\theta }\left[\omega \right]C_{\mathrm{XY}}^{\theta }\left[\omega \right]}{1-\sqrt{1-T_{2}}C_{\mathrm{YY}}^{\theta }\left[\omega \right]}},\\ \Xi_{Y}^{\prime }\left[\omega \right]= & -\frac{\frac{T_{2}\sqrt{1-T_{2}}C_{\mathrm{YX}}^{\theta }\left[\omega \right]C_{\mathrm{XY}}^{\theta }\left[\omega \right]}{1-\sqrt{1-T_{2}}C_{\mathrm{YY}}^{\theta }\left[\omega \right]}+T_{2}C_{\mathrm{XY}}^{\theta }\left[\omega \right]}{1-\sqrt{1-T_{2}}C_{\mathrm{XX}}^{\theta }\left[\omega \right]-\frac{\left(1-T_{2}\right)C_{\mathrm{YX}}^{\theta }\left[\omega \right]C_{\mathrm{XY}}^{\theta }\left[\omega \right]}{1-\sqrt{1-T_{2}}C_{\mathrm{YY}}^{\theta }\left[\omega \right]}},\\ \Xi_{\xi }^{\prime }\left[\omega \right]= & \frac{\frac{\sqrt{T_{2}}\sqrt{1-T_{2}}C_{Y\xi }^{\theta }\left[\omega \right]C_{\mathrm{XY}}^{\theta }\left[\omega \right]}{1-\sqrt{1-T_{2}}C_{\mathrm{YY}}^{\theta }\left[\omega \right]}+\sqrt{T_{2}}C_{X\xi }^{\theta }\left[\omega \right]}{1-\sqrt{1-T_{2}}C_{\mathrm{XX}}^{\theta }\left[\omega \right]-\frac{\left(1-T_{2}\right)C_{\mathrm{YX}}^{\theta }\left[\omega \right]C_{\mathrm{XY}}^{\theta }\left[\omega \right]}{1-\sqrt{1-T_{2}}C_{\mathrm{YY}}^{\theta }\left[\omega \right]}}, \end{array}$$

where $C_{\mathrm{YX}}^{\theta }\left[\omega \right]=-C_{\mathrm{XY}}^{\theta +\pi /2}\left[\omega \right]$, $C_{\mathrm{YY}}^{\theta }\left[\omega \right]=-C_{\mathrm{XX}}^{\theta +\pi /2}\left[\omega \right]$, $C_{Y\xi }^{\theta }\left[\omega \right]=-C_{X\xi }^{\theta +\pi /2}\left[\omega \right]$. It can be easily verified that in the limit of $C_{\mathrm{XY}}^{\theta }\left[\omega \right]\to 0$, the coefficients reduce to those given in Equation (17). When the anti-squeezed component of the input field is included, the squeezing spectrum takes the form

(25) $$\begin{array}{cc} S_{{\hat{X}}_{\mathrm{out},2}^{\theta }}\left[\omega \right]= & \frac{1}{4}\left({\left|\Xi_{X}^{\prime }\left[\omega \right]\right|}^{2}+{\left|\Xi_{Y}^{\prime }\left[\omega \right]\right|}^{2}\right)+{\left|\Xi_{\xi }^{\prime }\left[\omega \right]\right|}^{2}\left(n_{\mathrm{th}}+\frac{1}{2}\right). \end{array}$$

**Figure 5:**
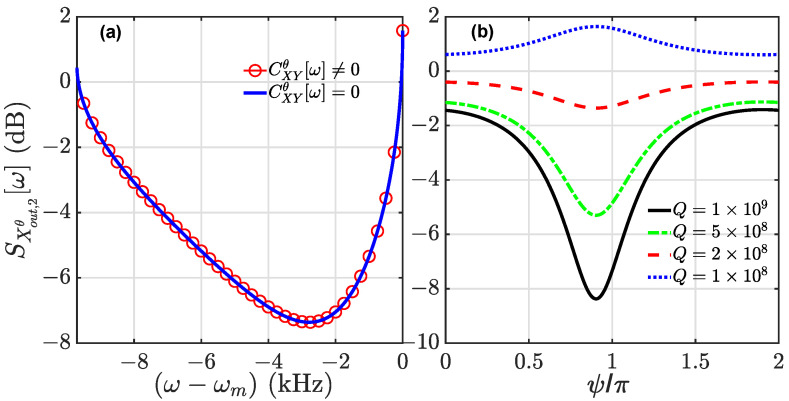
(**a**) Comparisons of squeezing spectra with $C_{\mathrm{XY}}^{\theta }\left[\omega \right]$ = 0 and $C_{\mathrm{XY}}^{\theta }\left[\omega \right]\neq 0$, and in (**b**) the squeezing spectrum is plotted for different values of the mechanical Q factors. Here, $T_{2}=0.75$, and the other parameters are the same as in Figure 2.

A comparison of the behavior of the squeezing spectra with and without $C_{\mathrm{XY}}^{\theta }\left[\omega \right]$ is shown in Figure 5a. It is clear that there is no difference between the spectra, indicating that there is no effect of the anti-squeezing component on the spectrum. This is because $C_{\mathrm{XY}}^{\theta }\left[\omega \right]$ is too small to change the squeezing spectrum. Thus, it is justified to neglect the influence of the anti-squeezing component of the input field $C_{\mathrm{XY}}^{\theta }\left[\omega \right]$ on squeezing of the output cavity field. Additionally, the squeezing is limited by the mechanical thermal noise under the optimal conditions in the above discussions. In Figure 5b, we plot the squeezing spectra for different values of the mechanical Q factor. The influence of the mechanical thermal noise is characterized by $n_{\mathrm{th}}\Gamma_{0}≃k_{B}T_{m}/\hbar Q$. Thus, high values of mechanical Q factor are necessary, since the squeezing is fragile to noise.

## 5. Conclusions

To conclude, we have investigated the possibility of enhancing squeezing in the output field of the cavity optomechancial system using a coherent feedback loop. We have shown that the optomechanical system operates under the situation of deamplification for the same generalized quadratures of input and output fields; the coherent feedback can effectively increase the squeezing level and bandwidth. For the fixed frequency, the optimal values of the transmissivity of BS and phase factor caused by time delays in the loop can be obtained, at which squeezing can be significantly enhanced. Moreover, optical losses due to the ineffective transmission in the feedback loop can degrade the squeezing because of the mixing of the uncorrelated vacuum noise. However, with the parameters of the current experiments, a significant enhancement in squeezing is still possible to achieve. We have also investigated the role of mechanical thermal noise, and found that high values of the mechanical Q factor are favorable for optical squeezing.

## Data Availability

Not applicable.
